# News Text Mining-Based Business Sentiment Analysis and Its Significance in Economy

**DOI:** 10.3389/fpsyg.2022.918447

**Published:** 2022-07-14

**Authors:** Ming Yang, Binghan Jiang, Yimin Wang, Tianyu Hao, Yuankun Liu

**Affiliations:** Faculty of Business and Economics, The University of Hong Kong, Hong Kong, Hong Kong SAR, China

**Keywords:** business sentiment analysis, text mining, convolutional neural network, economy, artificial intelligence

## Abstract

The purpose of business sentiment analysis is to determine the emotions or attitudes expressed toward the company, products, services, personnel, or events. Text analysis are the simplest and most developed types of sentiment analysis so far. The text-based business sentiment analysis still has some unresolved challenges. For example, the machine learning algorithms are unable to recognize double meanings, jokes and allusions. The regional differences between language and non-native speech structures cannot be explained. To solve this problem, an undirected weighted graph is constructed for news topics. The sentences in an article are modeled as nodes, and the normalized sentence similarity is used as the link of the nodes, which can help avoid the influence of sentence length on the summary results. In the topic extraction process, the keywords are not limited to a single word, to achieve the purpose of improving the readability of the abstract. To improve the accuracy of sentiment classification, this work proposes a robust news mining-based business sentiment analysis framework, called BuSeD. It contains two main stages: (1) news collection and preprocessing, and (2) feature extraction and sentiment classification. In the first stage, the news is collected by using crawler tools. The news dataset is then preprocessed by reducing noises. In the second stage, topics in each article is extracted by using traditional topic extraction tools. And then a convolutional neural network (CNN)-based text analyzing model is designed to analyze news from sentence level. We conduct comprehensive experiments to evaluate the performance of BuSeD for sentiment classification. Compared with four classical classification algorithms, the proposed CNN-based classification model of BuSeD achieves the highest F1 scores. We also present a quantitative trading application based on sentiment analysis to validate BuSeD, which indicates that the news-based business sentiment analysis has high economic application value.

## Introduction

Business sentiment analysis is to capture and track the opinions, emotions or feelings expressed by consumers when they are participating in various business interactions (Sun et al., 2020). Sentiment analysis involves the analysis and processing of text, natural language, computational linguistics, or biometrics. The purpose is to determine the emotions or attitudes expressed toward the company, products, services, personnel, or events. With the rapid development of the Internet, the boundary between information providers and users has become more and more blurred. Under the background of the Internet financial era, the sources and channels of financial information are becoming more and more rich and diverse. Investors have more channels to obtain relevant financial information. In the past century, financial research is often limited to the research of data in digital form, while ignoring the research of unstructured financial “data”. In the current Internet era, analysts’ research reports, stock bar forum posts the news of news media and unstructured text information such as microblog and Facebook can often reflect the investment sentiment of investors on the stock market. This information often plays a subtle role in investors’ investment decisions (Provoost et al., 2019).

Text-based sentiment analysis is a hot research topic in both business and artificial intelligence (Alantari et al., 2022). Text analysis are the simplest and most developed types of sentiment analysis so far. They are in great demand and have a long history of development. They are also the technology most used by enterprises and the public sector. Text-based sentiment analysis, especially for business purposes, can be summarized as dividing sentences, paragraphs, posts or documents into negative, neutral or positive categories. Among them, more complex emotion and attitude processing, meaning extraction, intention classification and emotion analysis based on linguistics are becoming more and more popular. Automatic emotion analysis is usually realized through supervised machine learning, dictionary based unsupervised mining or their combination (Veltmeijer et al., 2021). Crawling to popular public websites to extract news data is an efficient way of collecting data for business sentiment analysis.

A common concept of machine learning in sentiment analysis is that the success of sentiment artificial intelligence “training” always depends on the quality of the input data. Larger, better, and cleaner data sets are necessary to avoid the “garbage in, garbage out” situation (Sun et al., 2022). However, even we have high quality dataset, the text-based business sentiment analysis still has some unresolved challenges. For example, the machine learning algorithms are unable to recognize double meanings, jokes, and allusions. The regional differences between language and non-native speech structures cannot be explained. For sentiment intelligence, dealing with irony in written speech can be a difficult task, and there may be a distorted understanding of meaning and intention (Kim et al., 2021). Although social media is usually the source of opinions and intentions mined by machine learning algorithms, the language is undeniably specific and not necessarily real speech in real life (Zhang et al., 2022).

To improve the accuracy of business sentiment prediction, this work proposed a robust news mining-based business sentiment analysis framework, called BuSeD. It contains two main stages: (1) news collection and preprocessing, and (2) feature extraction and sentiment classification. In the first stage, the news is collected by using crawler tools. The news dataset is then preprocessed by reducing noises. In the second stage, topics in each article is extracted by using a classical topic extraction tool. An undirected weighted graph is constructed for news topics. The sentences in an article are modeled as nodes, and the normalized sentence similarity is used as the link of the nodes, which can help avoid the influence of sentence length on the summary results. In the topic extraction process, the keywords are not limited to a single word, to achieve the purpose of improving the readability of the abstract. And then a convolutional neural network (CNN)-based text analyzing model is designed to analyze news from sentence level. We conduct comprehensive experiments to evaluate the performance of BuSeD for sentiment prediction. Compared with state-of-the-art classification algorithms, the proposed CNN-based prediction model of BuSeD achieves the highest F1 scores. We also present a case study to validate the economic application of business sentiment analysis, which indicates that the news-based business sentiment analysis has high economic application value. The main contribution of this paper is as follows:

(1) We propose a CNN-based robust news mining-based business sentiment analysis framework. It can enhance the explainability of the regional differences between language and non-native speech structures by using the undirected weighted graph to model topics in sentences.

(2) We propose a CNN-based text sentiment analyzing model to improve the sentiment classification accuracy.

(3) We conduct comprehensive experiments to prove the efficiency of the proposed BuSeD.

The structure of the rest paper is as follows: Section 2 reviews the state-of-the-art works in text mining and business sentiment analysis. Section 3 presents the details of the proposed BuSeD framework. Section 4 introduces the experiment process and analysis the experiment results. Section 5 concludes this work and gives future research directions.

## Related Work

Using web crawling techniques and text mining methods, work (Pan et al., 2020) explored the impacts and mitigation measures of COVID-19 on China’s agricultural economy in a study of over 750,000 words on two media channels, WeChat and Sina Weibo, on COVID-19 and agriculture in China. Jo et al. (Jo and You, 2019) used text mining methods, relevant newspapers, and government documents to interpret South Korea’s announcement of a new measure. The purpose is to expand the National Health Insurance (NHI) coverage strategies and to explore the conflicts between them and the more active role the government should play in policy development. Work (Pejić Bach et al., 2019) examines what techniques are used in text mining, what is at the heart of the field and what data sources are commonly used for text mining in the financial sector’s operations, particularly in the age of the internet, big data and social media. The aim of work (Lee and Hong, 2020) is to explore trends in blockchain technology through text mining analysis of patents and news articles, and to propose a blockchain policy agenda by capturing societal interests.

By using a case study research approach, work (Hu and Wang, 2019) analyses its synergistic approach to teaching and learning through a journalism translation module, while suggesting that through the integration of academic and professional communities, trainers of translators may have their own reflections on innovative teaching and learning strategies. Work (Kovalev, 2021) explores the dual influence of market and political pressures on journalists and the resulting characteristics of censorship and self-censorship in Russia, through which the conditions of the Russian media market are explored. The purpose of work (Schmeller, 2022) is to share a content analysis of the usability of a business news article for teaching business model concepts in a capstone business course. They suggested that professors must use innovative, empirical approaches to improve learning.

In work (Souma et al., 2019), deep learning approach using recurrent neural networks combined with long-short term memory units was used to train Thompson Reuters news archive data from 2003 to 2012 and to test predictive power on news archive data from 2013. In work (Mohan et al., 2019), the accuracy of stock price prediction is improved by collecting a large amount of time series data and analyzing it using a deep learning model in conjunction with relevant news articles. In work (Alonso et al., 2021), the different uses of sentiment analysis in fake news detection are examined, the most relevant elements and drawbacks are discussed, as well as the requirements which should be met soon. Work (Štrimaitis et al., 2021) uses three supervised machine learning models for sentiment analysis of financial background news collected from Lithuanian language websites. Hyperparameter optimization is also performed using grid search to obtain the optimal hyperparameters for each classifier.

The above-mentioned methods do not solve the problem that it is not easy to explain the regional differences between language and non-native speech structures. In addition, the sentiment classification accuracy should be further improved. Therefore, in this paper, we propose a new method to enhance the regional differences between language and non-native speech structures, and to improve the classification accuracy.

## Business Sentiment Analysis by Analyzing News

**FIGURE 1:**
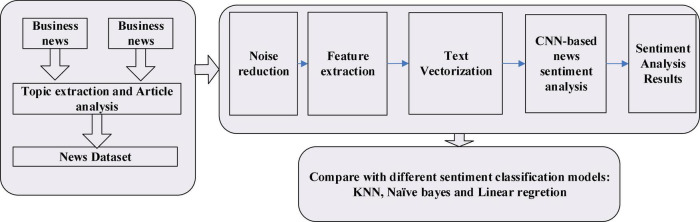
The workflow of BuSeD for sentiment analysis and economic decision making.

We propose a news mining and sentiment analysis framework, called BuSeD, to analyze news business sentiment, which can help make economic decisions. It contains two main parts. At first, the news is collected by using crawler tools. The news dataset is preprocessed by reducing noises. Topics in each article is extracted by using traditional topic extraction tools. And then a CNN-based text analyzing model is designed to analyze news from sentence level. The workflow of BuSeD is shown in Figure 1.

### Noise Reduction and News Topics Extraction

The news is first extracted by using the general-purpose web crawler (GPWC). GPWC is the whole web crawler. The implementation process is as follows. First, get the initial URL. The initial URL address can be manually specified by the user or determined by one or more initial crawling web pages specified by the user. Second, crawl the page according to the initial URL and get a new URL. After obtaining the initial URL address, you need to crawl the web page in the corresponding URL address first, then store the web page in the original database, find a new URL address while crawling the web page, and store the crawled URL address in a URL list for de duplication and judging the crawling process. Third, put the new URL into the URL queue. After obtaining the next new URL address in the second step, the new URL address will be put into the URL queue. Fourth, read the new URL from the URL queue and crawl the web page according to the new URL. At the same time, get the new URL from the new web page and repeat the above crawling process. Fifth, stop crawling when the stop conditions set by the crawler system are met. When writing crawlers, corresponding stop conditions are usually set. If the stop condition is not set, the crawler will crawl until the new URL address cannot be obtained. If the stop condition is set, the crawler will stop crawling when the stop condition is met.

Gensim is used to extract news topics. The input of Gensim is original and unstructured digital text (plain text). The semantic structure of the document is automatically found by calculating the statistical cooccurrence mode in the training corpus. These algorithms are unsupervised, which means that there is no need for manual input - just a set of plain text corpus. Once these statistical patterns are found, any plain text (sentence, phrase, word) can be expressed concisely by semantic representation. An undirected weighted graph is constructed for news topics. The sentences in an article are modeled as nodes, and the normalized sentence similarity is used as the link of the nodes, which can help avoid the influence of sentence length on the summary results. In the topic extraction process, the keywords are not limited to a single word, to achieve the purpose of improving the readability of the abstract.

Although business news text has the advantages of strong professionalism and low noise, the “noise interference” caused by massive text data will seriously affect the accuracy of the model. Noise interference means that noises in speech signals can disturb the communication connection between people. The reason is noises can cover up the acoustical components to hide information from listeners. The way to reduce the impact is to remove non-technical nouns and some unimportant prepositions in the original news text. In addition, the research collects common stop words for English and some meaningless digital symbols that may appear in news texts. It also introduces filtering processing to increase the impact of emotional words and verbs, which represent the emotions and views of financial stakeholders on the model.

We use the method of k-means clustering to filter noises in the news texts. Clustering is to group a data set into multiple groups or clusters. The data in the same cluster are highly similar, while the data in different clusters are quite different. Typical clustering algorithms include k-means (K-mean) and k-medoids (k-center point) algorithms. K-means-based clustering can realize noise cleaning. It treats the data grouped in smaller clusters as noise. It mainly uses the distance-based noise recognition method. Cluster analysis is carried out on the data set based on the distance between two data.

### Convolutional Neural Network-Based Fine-Grained Sentiment Analysis

A CNN-based text analyzing model is designed to analyze text sentiment from fine-grained sentence levels. The CNN is composed of an input layer, a convolutional layer, a pooling layer, a fully connected layer, and an output layer. The input is a convolution layer with only one feature surface. Its special network structure enables it to capture small feature information. This technique has been widely used in the field of image recognition. The parameter setting of the proposed CNN model is shown in Table 1.

**TABLE 1:** Structure of the CNN model for business sentiment analysis.

Layers	Cell number	Filter size
Convolution layer 1	150	(150,4)
Convolution layer 2	80	(80,4)
Pooling layer	80	–
Dense	10	Unit(10)

The input (embedding) layer converts the text into a vector and expands the dimension to meet the parameter requirements of the convolutional neural network. Then the convolutional layer and pooling layer are used to obtain local features and get the most important features. The convolutional layer is connected to the upper-layer feature surface through local connection and uses the feature of weight sharing to reduce the complexity of the model. The pooling layer uses max pooling to extract the most important features. During the training process, the Rectified Linear Unit (ReLU) is used as the activation function to transform the linear neural network into a nonlinear neural network. The output result is changed from *h*(x) in Formula 1 to Γ_*k*_(*x*) in Formula 2, which can efficiently speed up the convergence.


(1) $$h\,\left(\text{x}\right)=w^{\text{T}}\,x+b$$

(2) $$\Gamma_{k}\,\left(x\right)=\max\left(w^{\text{T}}\,\text{x}+b,0\right)$$

where h(x) represents the output of the linear output layer. Γ_*k*_(*x*) is the ReLU activation function to transform h(x) to non-linear form. k represents the kth convolution window, *w*^T^ is the weight matrix, and b is the bias value.

The corresponding output feature surface size after convolution and pooling operations is done by Formula 3.


(3) $$\phi_{i}=\frac{1}{W_{s\,z}}\,\left(\frac{\gamma_{s\,z}-\mu^{{}^{\prime }}}{\tau }+1\right)$$

where ϕ_*i*_ is the size of the output feature surface. γ_*sz*_ represents the size of each input feature surface. μ∈′[6,7,10] is the size of the convolution kernel. τ represents the sliding step size of the convolution kernel on the upper layer, and *W*_*sz*_ is the size of the pooling window. The model is trained by adjusting the number of parameters of the convolutional layers so that the size of ϕ_*i*_ is an integer.

The features trained by the convolutional layer and the pooling layer are used as the input and output classification results of the fully connected layer. That is, according to the probability distribution of sentences in different categories, output sentiment level labels for each sentence. o()i is the output of the text on the ith emotional tendency, which represents the probability that the text is classified as the ith emotional tendency. After normalization by softmax, o()i is expressed as:


(4) $$o\,\left(\sigma_{\text{i}}\right)=\frac{\text{exp}\,\left(\theta_{\text{i}}\cdot \text{v}+b_{\text{i}}\right)}{\sum_{j=1}^{n}\text{exp}\,\left(\theta_{\text{j}}\cdot \text{v}+b_{\text{j}}\right)}$$

where θ_*i*_ and b_i_ are the parameters and biases of the fully connected layer corresponding to the output σ_*i*_, respectively, and n is the total number of output categories.

To avoid over-fitting, dropout is used to make the features learned by neurons more robust. In addition, the model also limits the two-normal form size of the weight vector to achieve better classification results. In terms of measuring the prediction effect, this model uses cross entropy as the loss function. We apply CNN to text sentiment analysis, so the CNN model finally outputs sentiment classification results according to four rating scales, which are extremely negative, negative, positive and extremely positive.

## Experiment Analysis

### Experiment Data and Settings

We crawl more than 10,000 business news in recent five years from the Web. The training set data consists of 10,000 randomly selected finance and economics related news and 500 automatic abstracts of news retrieved with “business” as the keyword. The news from January 2018 to December 2021 were randomly selected according to the same proportion to ensure that the style of the media reports did not affect the training results. Each sentence was manually annotated, and a total of 80,000 sentences were annotated. The test set consists of 3,000 articles, which are formed by excluding 7,000 news articles selected as the training set from the collected 10,000 news articles and adopt the same labeling method as the training set. The evaluation criteria for sentiment analysis results in this paper are Precision, Recall and F1-Score (Sun et al., 2021).

### Compared Sentiment Analysis Models

To accurately analyze the sentiment in news data, other machine learning methods have also been tried in this work. Naive Bayes is a classification method based on Bayes’ theorem and the assumption of independence of feature conditions, and it is also one of the popular and popular classification models. Because NB needs to estimate few parameters, it is not sensitive to missing data, and the algorithm is relatively simple. In theory, the NB model has the smallest error rate compared with other classification methods, so it can be used as one of the candidate models for comparison of results.

Logistic Regression (LR) (Mehrolia et al., 2021), also known as logistic regression analysis, is a generalized linear regression analysis model, which is often used in data mining, automatic disease diagnosis, economic forecasting, and other fields. The independent variables can be either continuous or discrete. Through Logistic regression analysis, the weights of independent variables can be obtained to automatically perform features choose.

Support Vector Machine (SVM) (Zhou et al., 2021) is a commonly used discrimination method. In the field of machine learning, it is a supervised learning model that is usually used for pattern recognition, classification, and regression analysis. After researching many literatures, we found that many scholars now prefer to choose SVM for modeling analysis. Based on this, it is also used as one of the candidate models here.

KNN neighbor algorithm, or K-Nearest Neighbor (KNN) classification algorithm (Khorshid and Abdulazeez, 2021). It is one of the most effective and simple methods in data mining classification technology. The so-called K nearest neighbors is the k nearest neighbors, which means that each sample can be characterized and characterized by its nearest k neighbors. The core idea of the KNN algorithm is that if most of the k nearest neighbors of a sample in the feature space belong to a certain category, then the sample also belongs to this category and has the characteristics of the samples in this category. This method only decides the category of the sample to be classified according to the category of the nearest one or several samples in the decision of outputting the classification result. The KNN method is only related to a very small number of adjacent samples when making class decisions. Moreover, this method mainly uses the limited surrounding samples, rather than the method based on the discriminative class domain, to determine the class to which it belongs. Therefore, the KNN method is more suitable than other methods for the sample set to be divided with more overlapping or overlapping of class domains.

Dynamic convolutional neural network (DCNN) (Kalchbrenner et al., 2014). It integrates the dynamic k-max pooling.

One dimension CNN (1D-CNN) (Kim, 2014). It pretrains the word embeddings based on large amounts of news texts. It also adopts optimization strategy for fine-tuning.

Methods of processing text into word vectors include TF-IDF, Out-of-Dict, Word-Frequency, One-Hot, Word-Frequency and Google’s Word2Vec.

### Performance Comparison Between BuSeD and Classical Algorithms

We first analyze the high-frequency words of 10,000 news articles. After the stop words are removed, the top 20 words in the number of occurrences are shown in Table 2. As can be seen from Table 2, the high-frequency words are concentrated on the objects of the business and economics.

**TABLE 2:** Topics extracted from the crawled news.

Words	#	Words	#	Words	#	Words	#
Money	1200	Profitability	563	National	197	Countries	124
Opportunity	987	Political	338	Entrepreneur	188	Investment	130
Economic	933	International	372	Business plan	244	Power	270
Trade	670	Marketing	468	Business daily	278	Government	256
Income	489	Global	270	Financial news	345	Policy	148

Table 3 shows the comparison of BuSeD, LR, SVM, KNN, DCNN, and 1D-CNN in terms of precision, recall and F1-score. Compared with the four classical models, the proposed BuSeD achieves the optimal performance. The precision, recall and F1 of BuSeD are 0.968, 0.924, and 0.933 respectively, which are much higher than the values of LR, SVM and KNN.

**TABLE 3:** Comparison of sentiment classification results.

Methods	Precision	Recall	F1-Score
BuSeD	0.968	0.924	0.933
LR	0.877	0.862	0.843
SVM	0.923	0.894	0.903
KNN	0.912	0.894	0.908
DCNN	0.872	0.826	0.855
1D-CNN	0.928	0.915	0.920

### Validity Analysis of BuSeD

To further verify the validity of the sentiment model, a quantitative trading application based on sentiment analysis is analyzed. The application shows the correspondence between the real-time stock price data trends and the subject sentiment classified by BuSeD. Our study shows that when the stock price keeps rising, the sentiment of the financial news corresponding to the market should be positive. When the stock price keeps falling, the sentiment of the news corresponding to the market should be negative. Based on this hypothesis, we will try to verify the validity of the sentiment model BuSeD by the trend change of stock price and the synchronous change of news sentiment.

The stock price trend is mainly analyzed and predicted in terms of the stock price trend, news popularity and public opinion analysis at runtime. In terms of stock price trends, the study obtained the daily high/low and opening/closing prices of official stock prices through multiple channels and customized the corresponding stock price curve and box charts. In terms of news popularity, the research counted the news popularity of Hang Seng Index constituent stocks within 8 years and set up real-time follow-up on each day. In terms of public opinion analysis, the opinions, and sentiments of financial stakeholders on the stock price trend are extracted based on the BuSeD model.

The research uses the financial public opinion analysis module to analyze the relationship between news sentiment and stock price. Financial public opinion analysis refers to using BuSeD to analyze the sentiment polarity of news, and then infer the state of the market and the future stock price trends based on the direct relationship between the number of positive and negative news and stock price fluctuations. Through analysis, there is a strong correlation between news public opinion and stock price. When the positive news increases sharply or the negative news accumulates sharply, the market is in a turning period, and the stock price also moves on the corresponding direction.

## Conclusion

To improve the accuracy of sentiment classification, we proposed a robust news mining-based business sentiment analysis framework, called BuSeD. It has two main components: news collection and preprocessing, and feature extraction and sentiment classification. In news collection and preprocessing component, the news is collected by using crawler tools. The news dataset is then preprocessed by reducing noises. In feature extraction and sentiment classification component, topics in each article is extracted by using traditional topic extraction tools. And then a convolutional neural network (CNN)-based text analyzing model is designed to analyze news from sentence level. We conducted comprehensive experiments to evaluate the performance of BuSeD for sentiment classification. Compared with four classical classification algorithms, the proposed CNN-based classification model of BuSeD achieves the highest F1 scores. We also presented a quantitative trading application based on sentiment analysis to validate BuSeD, which indicates that the news-based business sentiment analysis has high economic application value.

## Data Availability Statement

The original contributions presented in this study are included in the article/supplementary material, further inquiries can be directed to the corresponding author/s.

## Author Contributions

MY proposed the idea and wrote the manuscript. BJ discussed with MY for proposing the idea and writing the manuscript. YW collected dataset and conducted the experiment. TH helped to collect dataset and provided suggestions in revision. YL helped to coding. All authors contributed to the article and approved the submitted version.

## Conflict of Interest

The authors declare that the research was conducted in the absence of any commercial or financial relationships that could be construed as a potential conflict of interest.

## Publisher’s Note

All claims expressed in this article are solely those of the authors and do not necessarily represent those of their affiliated organizations, or those of the publisher, the editors and the reviewers. Any product that may be evaluated in this article, or claim that may be made by its manufacturer, is not guaranteed or endorsed by the publisher.
